# Partial Characterization of the Impact of Saffron on the Sensory and Physicochemical Quality Traits of Dry-Cured Ham

**DOI:** 10.3390/foods10071506

**Published:** 2021-06-29

**Authors:** Elena M. Gómez-Sáez, Natalia Moratalla-López, Gonzalo L. Alonso, Herminia Vergara

**Affiliations:** 1 Benibaldo, S.A.U., 02007 Albacete, Spain; Calidad@benibaldo.com; 2 Cátedra de Química Agrícola, ETSI Agrónomos y de Montes, Campus Universitario, Universidad de Castilla-La Mancha, 02006 Albacete, Spain; Natalia.Moratalla@uclm.es (N.M.-L.); Gonzalo.Alonso@uclm.es (G.L.A.); 3 Science and Agroforestry Technology and Genetic Department, Higher Technical School of Agricultural Engineering and Forestry, Food Quality Section-Regional Development Institute, University of Castilla-La Mancha, 02006 Albacete, Spain

**Keywords:** ham, slices, *Crocus* *sativus* L., pH, color, sensorial quality, safranal

## Abstract

This study determined the effect of adding three concentrations of saffron (A: high, B: medium, and C: low) on vacuum-packaged dry-cured ham slices. The pH and the color coordinates were assessed at 0, 7, 14, 28 and 60 days of storage, and sensorial quality (visual appearance, odor and flavor) and safranal content were analyzed at 7, 14, 28 and 60 days. Saffron concentration did not significantly affect the pH or color (except in a* (redness) and b* (yellowness) at day 28; *p* < 0.001). Storage period affected pH values (*p* < 0.001) in all groups with a significant decline from day 28 (*p* < 0.05); the color coordinates showed a high stability (only L* (lightness) varied in the C group samples; *p* < 0.01). Sensorial quality did not vary with the time in any group. Significant differences were found among groups in visual appearance (*p* < 0.05) and flavor (*p* < 0.001) at day 14 and in odor at day 14, 28, and 60. In general, the C group samples obtained the highest scores. Safranal content varied significantly with the time in a different way in each group, with differences among groups at day 14 and 60 (*p* < 0.001).

## 1. Introduction

Spain ranks second in the European Union as regards pork production (4530,480–24,075,087 t) [1]. Among Spanish pork products, the most popular is the dry-cured ham (Jamón), the consumption per capita of which exceeds 1.60 kg [2]; it is typically offered as cured ham slices sold in trays owing to the increasing consumer demand for ready-to-eat products [3]. Dry-cured ham is a meat product highly appreciated by consumers because of its sensory characteristics made with pig hind limbs processed under traditional practices [4] that include salting, washing, draining, drying, and curing. This meat product is available under four official labels: “Jamón Serrano Traditional Specialty Guaranteed (TSG)”, “Jamón de Trévelez Protected Geographical Indication (PGI)”, “Jamón de Serón Protected Geographical Indication” and “Jamón de Teruel Protected Designation of Origin (PDO)”.

Meat product quality is determined by physicochemical, sensory, and hygienic-sanitary properties [5], and many factors can affect these parameters in dry-cured ham, such as raw material [6] or processing technologies [7] such as salting [8] cutting [9], and drying, which has an effect on texture [10].

Seasoning is used to aromatize meat products and make them safe from a microbiological and physicochemical perspective [11,12]. Unlike other Spanish meat products [13,14], which are manufactured with the most popular spices (white and black pepper, garlic, and paprika), dry-cured ham is typically seasoned only with salt. Other additives are sometimes used, such as sugar, antioxidants (E-301), preservatives (E-250 and E-252), and acidity corrector (E-331iii). However, saffron (the dried stigmas of *Crocus sativus* L.), one of the most important flavoring spices in Spain, has not yet been used. Some studies confirm that saffron alleviates inflammatory diseases such as diabetes [15] and cardiovascular diseases [16] and has preventive effects on cancer [17,18]. Saffron is composed of a group of carotenoids, crocetin sugar esters, picrocrocin, and a wide array of ketones and terpenic aldehydes, with safranal being the most important compound [19,20,21,22,23] that contributes to more than 70% of the aroma of Spanish saffron [22]. Safranal (2,6,6-trimethyl-1,3-cyclohexadiene-1-carboxaldehyde), which results from the hydrolysis of picrocrocin [24], is credited with specific bioactive effects, such as satiety-inducing, antidepressant, and neuroprotective effects [19,25] and protective effects on ischemia-induced PC12 cell injury through inhibition of oxidative stress and apoptosis [26]; safranal may also be used in future research on the treatment of schizophrenia [27]. 

Currently, saffron, which its use dates back to the Sumerians [28], is added to the main food dishes in different Mediterranean countries [28] as a natural food additive for coloring and flavoring [29], without limitation in culinary purposes (Regulation (EC) No 1333/2008 of the European Parliament and of The Council of 16 December 2008 on Food Additives). Saffron has no toxic effects when is used in culinary quantities [30].

To date, there are no reports on the use of saffron to seasone meat products such as dry-cured ham, however, saffron has been used to flavor cheese [31,32] and yoghurt [33]. When saffron is used to enhance the flavor of foods, it is used in very small concentrations so as not to detract from the flavor of the main product. Therefore, this study was carried out to investigate the effect of adding low concentrations of saffron by impregnation of sliced cured ham on the sensorial acceptance and physicochemical quality during the storage period. In addition, the transfer of aromatics from saffron to the product was assessed by analyzing the safranal content using headspace-stir bar sorptive extraction–gas chromatography/mass spectrometry (HS-SBSE–GC-MS).

The results of this study will contribute to the meat industry through the discovery of innovative products that may provide added value and have favorable health effects on consumers.

## 2. Materials and Methods

### 2.1. Experimental Design

In this study, 10 dry-cured hams (8 ± 1 kg and pH > 5.6)—from 5 Duroc female pigs—belonging to the official label “Jamón Serrano TSG” were used. The pigs were raised under intensive conditions and in compliance with animal welfare standard [34]. Transportation of hams from the slaughterhouse and cutting rooms to the manufacturing industry (provider of Benibaldo S.A.U., Albacete, Spain) was conducted in refrigerated vehicles at a temperature <3 °C. Then, the dry-cured hams were processed using the following protocol: hams were pitted, peeled, polished, knocked out, and sliced in a slicer (Model USA-280, José Bernad, S.L., Albacete, Spain). The slices (0.8 ± 0.1 mm thickness) were placed on a coating base until 100 g was reached. 

Because there are no previous studies on the addition of saffron to meat products, to establish the concentrations of this spice in each group, first, a preliminary sensory analysis was performed using a triangle test, to understand whether panelists can differentiate between the visual appearance of samples spiced with the lowest saffron concentration (0.015% *w*/*w*) and samples without saffron (control group). A sensory analysis was conducted following the recommendations made in a previous study [35], and the results were statistically analyzed according to [36]. According to a previous study in which 30 panelists participated in such a sensory analysis, the minimum number of correct answers for determining a perceptible difference should be 19 (∝ = 0.1%). In the present study, 28 of 30 panelists answered correctly. Thus, this concentration of saffron was considered the lowest that should be added to the ham slices. Therefore, the following groups were compared: A (high: 0.055% *w*/*w*), B (medium: 0.035% *w*/*w*) and C (low: 0.015% *w*/*w*) and a control group without saffron.

Ground saffron, under the PDO label “Azafrán de La Mancha” was directly purchased from a producer (Agrícola Técnica de Manipulación y Comercialización, Minaya, Albacete, Spain). Generally, this product is commercially available in stigma form and not in powder form. Ground saffron was characterized according to ISO 3632:2011 [37] ($A_{1\;cm}^{1\%}$ 440 nm = 230 ± 2, $A_{1\;cm}^{1\%}$ 257 nm = 95 ± 3, and $A_{1\;cm}^{1\%}$ 330 nm = 24 ± 1). Saffron was evenly added to the samples using a stainless-steel dredger (Model KCFINE, Kitchen Craft, 7.3 × 7.3 × 9.1, 140 g, Amazon, Spain). The temperature during the manufacturing process did not exceed 15 °C. Samples (sachets of ham slices of 100 g each) were packed under vacuum conditions with a packaging machine (Model JB-350/M, José Bernad, S.L.) using a base to plate ham (Model 16409, 26 cm, Manchaplas, S.L., Albacete, Spain) and vacuum bags (Model 90M, 350 × 300 mm^2^, Gutplask, S.L., Getafe, Madrid, Spain) with an oxygen permeability rate <70 cm^3^/m^2^/24 h, tensile strength at break of 21–43 MPa, elongation at break of 400–600%, and a slow resistance to penetration >1 N. After packaging, samples were stored in the dark at 2 °C until the analysis. Physicochemical quality was analyzed at 0, 7, 14, 28, and 60 days of storage, whereas the sensory analysis was done after 7 days of preparation. A total of 192 sachets were prepared, of which 20 and 160 were used in the physicochemical and sensory analysis, respectively, and 12 were used to analyze the transfer of aromatics.

### 2.2. Analysis of Samples

#### 2.2.1. Physicochemical Quality (pH and Color Parameters)

To determine pH values, a pH meter (Crison GLP 22 + pH & Ion-meter-Crison Instruments, S.A., Barcelona, Spain) connected to a penetration electrode was used. pH was directly measured on five different slices randomly selected from each sachet. 

Color coordinates (L*, lightness; a*, redness; and b*, yellowness) were evaluated using a CR 400 chroma meter (Minolta, Osaka, Japan) with a D65 illuminant and 10° standard observer, calibrated against a standard white tile. In all ham groups, five measurements were randomly taken on the surface of the sample on each sachet, and the mean value of three measurements was used. Chroma [C* = (a^2^ + b^2^)^1/2^] and hue angle (h* = tan^−1^ (b*/a*)° were calculated [38].

#### 2.2.2. Sensorial Quality

To measure the degree of acceptance or rejection of the three groups of flavored ham, a hedonic test was performed at 7, 14, 28, and 60 days of storage, at mid-morning in the test room of the university for 45 minutes approximately. It was carried out by 30 panelists (the same ones who participated in the triangular test described above; regular consumers of dry-cured ham; between 20 and 70 years old, 48% women, belonging to the university community). The attributes to evaluate were: Visual appearance: color assessment relating to the red color and presence of saffron. Odor: assessment relating to the characteristic odor associated with curing process and mixed with saffron. Flavor: assessment relating to the characteristic taste associated with the salt and curing process mixed with saffron. Samples were kept at environmental temperature for half an hour before the tasting. Three flavored dry-cured ham slices, one from each group, were placed in plastic plates and codified with three random numbers. Cold water and toasted bread were supplied to each panelist before testing each sample for cleansing the palate. Panelists, untrained consumers, were instructed at the beginning of each session for 15 minutes. The test they were to perform and how to proceed after eating each slice of flavored ham was explained to them.

The samples were rated on a 5-point hedonic scale, as follow: 1 = “Do not like it”, 2 = “Slightly dislike it”, 3 = “Neither like it nor dislike”, 4 = “Like it” and 5 denoted “I like it very much”. The consumers chose the expression in relation to their perception and acceptance of the flavored group. Then, the panelists indicated the concentration they liked the most overall.

#### 2.2.3. Analysis of Safranal in Dry-Cured Ham

The transfer of aromatics from saffron to the meat product—flavored dry-cured ham with this spice—was analyzed by HS-SBSE–GC-MS. The volatile compounds were desorbed from a polydimethylsiloxane-coated stir bar (0.5 mm film thickness × 20 mm length; Twister, Gerstel GmbH (Mülheim an der Ruhr, Germany) using an automated thermal desorption unit (TDU, Gerstel) mounted on an Agilent 7890A gas chromatography system coupled to a quadrupole Agilent 5975C electron ionization mass spectrometric detector (Agilent Technologies, Palo Alto, CA, USA) equipped with a fused silica capillary column (BP21 stationary phase; 30 m length, 0.22 mm internal diameter, and 0.25 µm film thickness; SGE, Ringwood, Australia). The carrier gas was helium with a constant column pressure of 20.75 psi. From each group, 200 mg of flavored dry-cured ham was used (every sachet was divided into four equal parts and 25 mg from each part was used) for each time point (7, 14, 28, and 60 days of storage). These 200 mg were analyzed in triplicate to detect and quantify the major component of saffron (safranal), which is the main compound that can be used to distinguish and classify cured ham flavored with saffron [22]. Thus, 36 vials of 10 mL were used, and the method validated in a previous study [22] was used to analyze the transfer of aromatics from saffron to dry-cured ham.

Mass spectrometry data acquisition was performed in the positive scan mode; however, to avoid matrix interferences, the MS quantification was performed in the SIM mode using the major ion of safranal.

### 2.3. Statistical Analysis

Data were analyzed using the statistical package SPSS 24.0 version (SPSS Inc., Chicago, IL, USA). To analyze the effect of saffron concentration (A: high, B: medium, and C: low) on the physicochemical parameters (pH and color), sensorial quality (visual appearance, odor, and flavor), and safranal transfer, a Shapiro-Wilk test was carried out to check the normality and a Levene’s test of homogeneity of variance of all values, then, a one-way analysis of variance (ANOVA) was performed. Moreover, within each group, ANOVA was performed to check the effect of storage time. When the differences were statistically significant (*p* < 0.05), a Tukey’s test was carried out to identify differences between pairs of groups. Correlation between safranal and the sensorial and physicochemical parameters was determined by estimating Pearson correlation coefficients.

## 3. Results 

### 3.1. Physicochemical Quality (pH and Color Parameters)

Table 1 shows the pH and color parameters of each group (control, A: 0.055%, B: 0.035%, and C: 0.015% *w*/*w*), and the changes in these values in the dry-cured ham slices during the storage period (0, 7, 14, 28, and 60 days). Throughout the storage period, pH values ranged from 5.96 to 5.42. From day 7, there were no significant differences among groups. In all samples, a gradual decrease in pH was observed with storage time, with significant differences between groups from 28 days of storage. 

L* values were similar in all groups, and no statistical differences were found among samples at any time during storage. Notably, both in the control samples and in the flavored sample with the lowest saffron concentration (C), this parameter gradually decreased until 28 days of storage and then increased significantly. However, the L* values showed high stability in the A and B groups.

**Figure 1:**
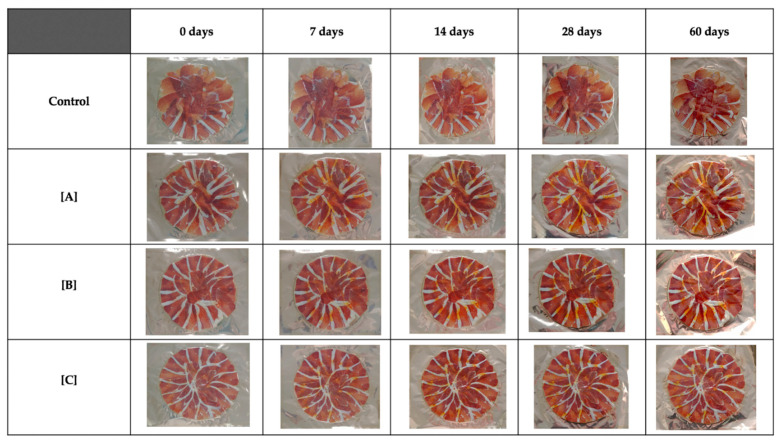
Visual appearance of the samples in each group (CONTROL: sample without saffron; A: 0.055% *w*/*w*; B: 0.035% *w*/*w*; C: 0.015% *w*/*w*) during the storage time period (0, 7, 14, 28 and 60 days). Scale (1:20).

Redness, yellowness and Chroma did not vary with storage time. However, there was a significant difference due to the added saffron concentration at 28 days (*p* < 0.01). At this time point, the values of these color parameters followed the next order A ≥ B ≥ C ≥ control. In Hue (h°) these differences (*p* < 0.01) were observed at 28 days and at the end of the experiment and with the same above order. Huge angle showed a high stability in control and A groups. Visual appearance of the samples in each group during the storage time period is showed in Figure 1.

### 3.2. Sensorial Quality

Table 2 shows the score given by the panelists to the spiced samples from day 7 of storage to the end of the experiment (60 days). There were no differences due to the gender of the panelists, and the results showed great stability in all groups, with values always higher than 3 and close to 4, which indicate that the spiced ham is to the taste (visual appearance, odor, and flavor) of consumers.

**Figure 2:**
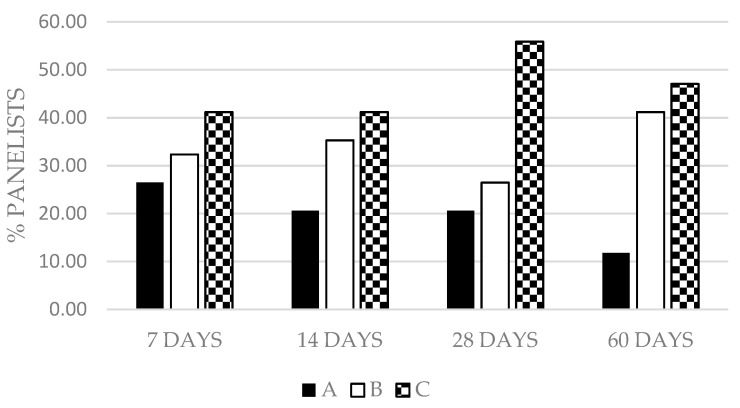
Percentage of panelists who considered a particular group favorite (A: 0.055% *w*/*w*; B: 0.035% *w*/*w*; C: 0.015% *w*/*w*) during the study period (7, 14, 28 and 60 days of storage).

Significant differences due to saffron concentration were observed in visual appearance (*p* < 0.05) and flavor (*p* < 0.001) at 14 days and in odor at 14, 28, and 60 days (*p* < 0.05 at 14 and 28 d; *p* < 0.01 at 60 days) with a similar trend for the three sensory parameters: C ≥ B ≥ A, depending on the time of analysis and the parameter. Figure 2 presents the percentage of panelists who considered a particular group favorite.

### 3.3. Transfer of Aromatic Compounds from Saffron

Safranal content and its trend throughout the experiment is shown in Table 3. Only at 14 and 60 days of storage, there were differences (*p* < 0.001) among groups, and the groups were ordered according to safranal content as A > B > C and A > B = C, respectively. Safranal content decreased from day 7 in all samples, but subsequently, the tendency was different in each group with significant differences (*p* < 0.001 in A and B; *p* < 0.05 in C). It is noteworthy that safranal content increased in all samples at 60 days. Correlation between safranal content and other parameters is shown in Table 4. Only there was a significant correlation (*p* < 0.05) with odor (*r* = 0.65) in group C.

## 4. Discussion 

### 4.1. Physicochemical Quality (pH and Color Parameters)

#### 4.1.1. pH

The pH values found in our study were similar to those reported in previous studies [39,40] in dry ham after a similar storage time. The decline in this parameter is in agreement with the findings of a previous study [41] on the effect of storage under vacuum conditions for 8 months on dry-cured ham quality. In contrast, another study [39] on the shelf life of sliced dry-cured ham packaged under vacuum with analysis performed in the same storage period as the present study reported an increase in pH during storage time. This increase has been associated with the release of amino acids and other basic compounds during the dry-maturation stage [42].

pH is an important factor influencing the growth of microorganisms, with low pH inhibiting the growth of pathogens [43]. However, there are pathogenic microorganisms such as *Listeria* *monocytogenes* that can grow in the pH range observed in this study affecting the ham quality [44]. Therefore, other factors may be crucial to prevent their growth, such as low water activity and maintaining sliced ham at refrigeration temperatures [45]. According to [46] for cured meat product, such as ham, to be considered stable during storage and distribution, one of the conditions is that the pH is less than 6.0. In our study, this parameter was lower than this limit in all groups.

#### 4.1.2. Color Parameters

Color is an important quality characteristic that contributes to the sensorial acceptability of dry-cured ham [47]. However, color is affected by many factors such as spices added, packaging or processing [48]. Changes in color parameters have been studied in dry ham [40,49,50,51,52].

The L* parameter has been associated with the thin layer of moisture on the muscle surface [53] and lightness in these muscles depends on the water content (moisture) and water movement (dehydration) towards the surface [42]. For some authors [47] is considered the most important parameter determining quality of meat products. According to [54] changes in this parameter in the sliced dry-cured ham could be negative since modifications in the typical color of dry-cured ham could influence consumers. It is evident that the addition of saffron with concentrations such as in A or B groups caused a high stability in lightness. Nevertheless, the results in C and control group were contrary to the results of authors such as [40,50] who determined that L* preserved color during similar storage time in sliced dry-cured ham.

According to [49], redness is used as an indicator of color stability while yellowness has been associated with rancidity. Authors such as [55] concluded that a* value was the most important aspect of color. In our study, the a* and b* parameters did not vary with storage time. Others [49] have also reported similar stability in a* and b* in ham slices after 8 weeks of storage in vacuum packaging. The obtained results could be attributed to the presence of crocetin esters, also known as crocins, a group of water-soluble carotenoids responsible for saffron’s color strength [23,28,56]. Crocetin is formed from crocins during storage time [57]. Due to the fact crocetin is fat-soluble, it could cause the yellowness to increase in dry-cured ham slices [58].

According to [59] the characterization of the color is achieved by means of the coordinates of L*, a* and b*, but the main purpose in the measurements of the color is the objective determination of their differences through the parameters of chroma (C*) and of the tone (h*). Our results showed that the addition of saffron gives a greater C* and tone to the ham slices, reaching significant differences among groups at 28 days in both parameters and at the end of experiment in hue.Authors such as [60] have studied the chroma and hue in Spanish saffron and dry-cured Duroc ham [61], but there are not previous references which had studied the color parameters of dry-cured ham flavored with this spice.

### 4.2. Sensorial Quality

Sensory evaluation started developing with the growth of industry and processed food [62]. Sensory characteristics are crucial in the development of new food products [63] and influence consumer acceptance both before purchase (visual appearance) and at the time of consumption (odor and flavor). Because of this, sensory analysis are one of the most important methods in judging food quality [64]. Previous studies [52,65,66,67,68,69,70] have reported these parameters in ham and indicated the importance of flavor in the overall quality of dry-cured ham. However, the present study is the first to our knowledge to determine the degree of satisfaction of cured ham spiced with saffron. The addition of spices provide new tastes, colors and aromas to food that even gives culinary identity [71], owing to the changes in the composition of volatile compounds [72] that affect the hedonic characteristics [73] and may affect the acceptance of new products [74]. On the other hand, spices could improve the quality of meat products due to their preservatives properties [75].

The addition of saffron provoked a great stability during time of study in each group. In this work, all groups were accepted by consumers. It is evident that the panelists preferred the group with the lowest concentration of saffron (Figure 2). Other studies [52,76] that indicated the acceptability of dry-cured ham during storage obtained lower scores with storage time, owing to increasing rancid odor and flavor in vacuum-packed ham [50,51,77]. Despite the fact the shelf life assigned to Spanish dry-cured ham is approximately one year, this is significantly reduced when the dry-cured ham is sliced and vacuum-packaged [51,78]. The decrease of flavor, odor and even color is in accordance with the reduction of shelf life of ham, not due to microbiological problems, but because of the decrease of sensorial quality [51]. This rancidity is usually associated with a decrease in pH [79] and especially in products rich in unsaturated fatty acids [80] such as ham. This may have occurred in the present study (note that we did not analyze lipid oxidation) and affect the scores of the panelists. However, these scores did not vary significantly during the experimental period, a finding that may be attributed to the addition of saffron, which may have masked the negative effect of lipid oxidation or decelerate it owing to its antioxidant power [81]. Significant differences due to saffron concentration could be attributed that safranal, the major aromatic component of saffron, changes over time increasing its concentration [82,83] affecting to hedonic characteristics.

### 4.3. Transfer of Aromatic Compounds from Saffron

Because safranal is one of the major components of saffron [84] and represents 72% of the flavoring composition of saffron [85,86], its content was determined to assess the transfer of aromatic compounds from the spice to the ham. Such saffron compounds were not found in the control group ham samples, which indicates that dry-cured ham and saffron do not have common aromatics. The amount of safranal contained in dry-cured ham was much lower (10^−7^) than the safranal content present in the spice itself [85]. This gives a subtle saffron flavor to the dry-cured ham without masking its origin flavor but enhancing it [28].

In all groups, there is a rapid decrease of 7 to 14 days, consistent with that detected by the panelists as shown in Table 2, and a different increase at each group to 60 days. These findings are consistent with previous findings [23,85] that indicated that safranal concentration is higher in saffron stored longer than a month because of formation of safranal from crocetin esters and picrocrocin during storage [28]. Previous studies reported that the main compounds of saffron change over time [82,83].

The method used to determine the transfer [22] only analyses the safranal in the surface layer of the slice. Therefore, as fat is a lipophilic medium that absorbs apolar substances [85], it causes a decrease in the safranal content of the such layers. However, during storage, the generation and the absorption of safranal compete, being the absorption process faster than the generation process. It could be due to the fact that the internal layers that have absorbed safranal became saturated with the compound generated after 28 days. This could occupy the surface layers, recovering the initial values of 7 days. It is shown in the evolution of A and C and the trend in B groups. Moreover, this is in agreement with the significant differences between groups at day 14 (with lower concentration of safranal) and 60 days (with higher concentration of safranal).

With decreasing concentration of saffron used to season the ham, the sensory scores improved, and the correlation changed from negative in group A to positive in group C (*r* = 0.30 with visual appearance, *r* = 0.34 with flavor, and *r* = 0.65 (*p* < 0.05) with odor). This agrees with the highest organoleptic scores obtained by group C samples (Table 2). Correlation between color parameters agrees with the previous results explained in Table 1, due to the change of the main saffron compounds during storage. Correlation of safranal content with pH (always negative) and with the color coordinates were not significant in any group.

## 5. Conclusions

The results of this study suggest that (1) the pH of ham decreases throughout storage, and (2) the color coordinates do not change over time even with the addition of saffron. (3) It is advisable not to use a saffron concentration higher than that used in group C because it negatively affects sensory acceptance. (4) The safranal content varies throughout storage and shows a positive correlation with sensory parameters, especially when saffron concentrations are lower. Future studies should analyze the effect of adding other spices to ham slices, to offer new meat products to consumers.

## Figures and Tables

**Table 1:** Effect of different added concentrations of saffron and storage period on the physicochemical characteristics (pH and color; mean ± s.e.) of sachets of ham slices of ham.

Parameters	Storage Period (Days)	Concentration				ANOVA
		CONTROL (n = 5)	A (n = 5)	B (n = 5)	C (n = 5)	
pH	0	5.96 ± 0.12 ^y, c^	5.95 ± 0.13 ^xy, c^	5.77 ± 0.05 ^x, b^	5.80 ± 0.10 ^xy, b^	*
	7	5.76 ± 0.16 ^b^	5.70 ± 0.03 ^ab^	5.73 ± 0.03 ^b^	5.68 ± 0.04 ^b^	NS
	14	5.71 ± 0.04 ^b^	5.73 ± 0.23 ^bc^	5.71 ± 0.07 ^b^	5.83 ± 0.25 ^b^	NS
	28	5.69 ± 0.09 ^b^	5.67 ± 0.02 ^ab^	5.77 ± 0.07 ^b^	5.69 ± 0.04 ^b^	NS
	60	5.48 ± 0.05 ^a^	5.48 ± 0.06 ^a^	5.42 ± 0.05 ^a^	5.44 ± 0.07 ^a^	NS
Effect of storage period		***	***	***	***	
L*	0	42.37 ± 5.08 ^ab^	45.05 ± 1.77	44.17 ± 7.08	47.28 ± 4.25 ^b^	NS
	7	48.65 ± 5.22 ^b^	49.15 ± 3.49	45.67 ± 3.44	45.02 ± 1.61 ^b^	NS
	14	41.64 ± 4.61 ^ab^	43.23 ± 5.67	45.54 ± 8.58	43.81 ± 4.61 ^ab^	NS
	28	35.32 ± 1.95 ^a^	40.94 ± 5.08	36.97 ± 4.63	35.55 ± 3.38 ^a^	NS
	60	43.61 ± 3.73 ^b^	45.45 ± 5.64	46.39 ± 6.34	46.09 ± 7.93 ^b^	NS
Effect of storage period		**	NS	NS	**	
a*	0	19.41 ± 2.98	23.83 ± 3.20	22.15 ± 5.93	18.89 ± 3.50	NS
	7	19.22 ± 1.63	20.38 ± 1.82	21.67 ± 2.57	20.68 ± 1.69	NS
	14	18.32 ± 2.97	20.00 ± 4.39	18.96 ± 5.73	20.48 ± 4.11	NS
	28	17.85 ± 0.77 ^x^	22.29 ± 2.21 ^y^	22.28 ± 1.73 ^y^	20.80 ± 2.38 ^xy^	**
	60	18.97 ± 2.10	17.13 ± 5.53	15.45 ± 7.34	16.24 ± 7.26	NS
Effect of storage period		NS	NS	NS	NS	
b*	0	21.34 ± 7.85	29.27 ± 7.26	23.38 ± 5.81	18.70 ± 3.74	NS
	7	22.99 ± 6.48	31.62 ± 9.69	28.38 ± 3.84	24.91 ± 4.23	NS
	14	20.47 ± 7.69	30.63 ± 6.65	30.65 ± 14.02	25.73 ± 10.12	NS
	28	12.37 ± 1.59 ^x^	28.40 ± 10.38 ^y^	20.93 ± 4.27 ^xy^	16.86 ± 6.32 ^xy^	**
	60	16.23 ± 6.52	29.83 ± 4.73	28.91 ± 10.23	26.20 ± 9.66	NS
Effect of storage period		NS	NS	NS	NS	
Chroma (C*)	0	29.00 ± 7.67	37.84 ± 7.32	32.66 ± 5.64	26.71 ± 4.22	NS
	7	30.22 ± 5.07	37.80 ± 8.95	35.73 ± 4.42	32.45 ± 3.79	NS
	14	27.63 ± 7.53	37.00 ± 4.98	36.62 ± 13.29	33.13 ± 9.94	NS
	28	21.75 ± 0.99 ^x^	36.37 ± 9.36 ^y^	30.65 ± 3.88 ^xy^	27.01 ± 5.46 ^xy^	**
	60	25.41 ± 4.39	34.68 ± 5.31	32.91 ± 12.16	31.33 ± 10.32	NS
Effect of storage period		NS	NS	NS	NS	
Hue (h*)	0	46.40 ± 6.72	50.35 ± 4.77	46.46 ± 10.99 ^a^	44.61 ± 6.02 ^ab^	NS
	7	49.23 ± 8.10	56.19 ± 5.79	52.61 ± 2.12 ^ab^	50.02 ± 4.39 ^ab^	NS
	14	34.66 ± 3.87	50.28 ± 8.06	42.86 ± 4.44 ^ab^	37.95 ± 7.81 ^ab^	NS
	28	46.81 ± 7.01 ^x^	56.26 ± 9.81 ^y^	55.78 ± 12.52 ^xy, a^	49.48 ± 8.86 ^x, a^	**
	60	39.18 ± 11.75 ^x^	60.49 ± 8.37 ^y^	63.29 ± 6.48 ^y, b^	57.60 ± 11.95 ^y, b^	**
Effect of storage period		NS	NS	**	*	

CONTROL: sample without saffron; A: 0.055% *w*/*w*; B: 0.035% *w*/*w*; C: 0.015% *w*/*w*. NS: No significant. *, **, ***, indicates significance levels at 0.05, 0.01 and 0.001, respectively. ^x,y^, values in the same row with different superscript are significantly different due to the group (CONTROL, A, B and C). ^a,b,c^, values in the same column with different superscript are significantly different due to the different storage period (0, 7, 14, 28 and 60 days). s.e.: standard error.

**Table 2:** Effect of different added concentrations of saffron on sensory characteristics (visual appearance, odor, and flavor; means ± s.e.) of sachets of ham slices.

Parameters	Storage Period (Days)	Concentration			ANOVA
		A (n = 30)	B (n = 30)	C (n = 30)	
Visual appearance	7	3.62 ± 0.85	3.74 ± 1.05	3.68 ± 0.98	NS
	14	3.42 ± 1.02 ^x^	3.71 ± 0.92 ^xy^	3.98 ± 0.84 ^y^	*
	28	3.71 ± 1.02	3.66 ± 0.97	3.91 ± 0.92	NS
	60	3.75 ± 0.84	4.00 ± 0.76	3.53 ± 0.97	NS
Effect of storage period		NS	NS	NS	
Odor	7	3.76 ± 0.82	3.76 ± 1.01	3.68 ± 0.81	NS
	14	3.53 ± 0.95 ^x^	3.80 ± 0.78 ^xy^	3.94 ± 0.87 ^y^	*
	28	3.60 ± 0.81 ^x^	3.57 ± 1.09 ^x^	4.17 ± 0.86 ^y^	*
	60	3.42 ± 1.27 ^x^	4.14 ± 0.87 ^y^	4.08 ± 0.77 ^y^	**
Effect of storage period		NS	NS	NS	
Flavor	7	3.50 ± 1.14	3.71 ± 0.87	3.79 ± 0.91	NS
	14	3.46 ± 1.08 ^x^	4.00 ± 0.82 ^y^	4.20 ± 0.72 ^y^	***
	28	3.74 ± 0.95	3.91 ± 1.01	3.91 ± 0.78	NS
	60	3.58 ± 0.94	4.00 ± 0.86	3.72 ± 0.85	NS
Effect of storage period		NS	NS	NS	

A: 0.055% *w*/*w*; B: 0.035% *w*/*w*; C: 0.015% *w*/*w*. NS: No significant. *, **, *** indicates significance levels at 0.05, 0.01 and 0.001, respectively. ^x,y^, values in the same row with different superscript are significantly different. 1: Do not like it; 2: I slightly dislike it; 3: Neither like nor dislike; 4: Like it; 5: I like it very much. s.e.: standard error.

**Table 3:** Determination of safranal (µg/ 100 g ham; means ± s.e.) in sachets of sliced dry-cured ham during the storage period.

Parameter	Storage Period (Days)	Concentration			ANOVA
		A (n = 3)	B (n = 3)	C (n = 3)	
Safranal	7	4.45 ± 0.31 ^b^	4.03 ± 0.18 ^b^	3.22 ± 0.40 ^b^	NS
	14	2.99 ± 0.12 ^z, a^	1.36 ± 0.01 ^y, a^	0.69 ± 0.07 ^x, a^	***
	28	2.89 ± 0.25 ^a^	1.33 ± 0.29 ^a^	3.00 ± 0.74 ^b^	NS
	60	5.46 ± 0.27 ^y, b^	2.08 ± 0.46 ^x, a^	2.57 ± 0.26 ^x, ab^	***
Effect of storage period		***	***	*	

A: 0.055% *w*/*w*; B: 0.035% *w*/*w*; C: 0.015% *w*/*w* saffron. NS: No significant. *, ***, indicates significance levels at 0.05 and 0.001, respectively. ^x,y,z^, values in the same row with different superscript are significantly different due to saffron concentration of saffron. ^a,b^, values in the same column with different superscript are significantly different for the different storage period (7, 14, 28 and 60 days). s.e.: standard error.

**Table 4:** Correlation coefficients between safranal content with the sensorial and physicochemical parameters in each group.

Group	Visual Appearance	Flavor	Odor	pH	L*	a*	b*	C*	h*
**A**	−0.13	−0.25	−0.16	−0.57	0.27	−0.26	0.06	0	0.24
**B**	0.12	−0.11	0.45	−0.03	0.31	0.12	0.53	0.4	0.35
**C**	0.3	0.34	0.65 *	−0.49	−0.18	0.18	−0.11	−0.02	−0.15

A: 0.055% *w*/*w*; B: 0.035% *w*/*w*; C: 0.015% *w*/*w*. *, indicates correlation significant at the 0.05 level.

## Data Availability

The data presented in this study are available from the corresponding author upon request.
